# Application of Starch, Cellulose, and Their Derivatives in the Development of Microparticle Drug-Delivery Systems

**DOI:** 10.3390/polym15173615

**Published:** 2023-08-31

**Authors:** Paolina Lukova, Plamen Katsarov, Bissera Pilicheva

**Affiliations:** 1 Department of Pharmacognosy and Pharmaceutical Chemistry, Faculty of Pharmacy, Medical University of Plovdiv, 4002 Plovdiv, Bulgaria; paolina.lukova@mu-plovdiv.bg; 2 Department of Pharmaceutical Sciences, Faculty of Pharmacy, Medical University of Plovdiv, 4002 Plovdiv, Bulgaria; bisera.pilicheva@mu-plovdiv.bg; 3 Research Institute at Medical University of Plovdiv, 4002 Plovdiv, Bulgaria

**Keywords:** starch, maltodextrin, cyclodextrin, cellulose, microparticles

## Abstract

Micro- and nanotechnologies have been intensively studied in recent years as novel platforms for targeting and controlling the delivery of various pharmaceutical substances. Microparticulate drug delivery systems for oral, parenteral, or topical administration are multiple unit formulations, considered as powerful therapeutic tools for the treatment of various diseases, providing sustained drug release, enhanced drug stability, and precise dosing and directing the active substance to specific sites in the organism. The properties of these pharmaceutical formulations are highly dependent on the characteristics of the polymers used as drug carriers for their preparation. Starch and cellulose are among the most preferred biomaterials for biomedical applications due to their biocompatibility, biodegradability, and lack of toxicity. These polysaccharides and their derivatives, like dextrins (maltodextrin, cyclodextrins), ethylcellulose, methylcellulose, hydroxypropyl methylcellulose, carboxy methylcellulose, etc., have been widely used in pharmaceutical technology as excipients for the preparation of solid, semi-solid, and liquid dosage forms. Due to their accessibility and relatively easy particle-forming properties, starch and cellulose are promising materials for designing drug-loaded microparticles for various therapeutic applications. This study aims to summarize some of the basic characteristics of starch and cellulose derivatives related to their potential utilization as microparticulate drug carriers in the pharmaceutical field.

## 1. Introduction

Much research nowadays is focused on formulating polymer microparticles as novel drug-delivery systems and providing modified drug release. Conventional formulations cannot offer controlled delivery of the therapeutic agents and are usually not associated with target specificity. That is why the development of methods to control the rate and site of drug release has gained serious popularity in recent years. Incorporation of active pharmaceutical ingredients into polymer microcarriers has been outlined as a successful strategy to create drug-delivery systems that enable drug release at a desired rate and extend. Controlled-release polymer microparticles can also increase drug stability, especially in molecules that are prone to rapid degradation in the body. Such formulations are generally associated with a significant reduction in the number of applications and better patient acceptance [1,2,3].

Many studies have reported that drug microcarriers can be successfully used as drug-delivery systems in the therapy of various diseases. The main challenges in their development are related to achieving stability, uniform particle size, controlling the rate and site of drug release, and scaling up. The rapid development in recent years in this area of drug technology is undeniable proof of the advantages of such microcarrier systems and the numerous possibilities they can offer [4,5,6,7].

Microparticles are structures ranging in size from 1 to 1000 μm. They are usually made of a polymer matrix in which the active substance is included. Depending on how the drug molecules are incorporated, microparticles are mainly of two types: microspheres (homogeneous mixture of active substance and polymer) and microcapsules (drug core coated with polymer) [8]. Historically, Holliday et al. were the first to patent the use of polymer microparticles in the pharmaceutical industry back in the 1970s [9]. They encapsulated acetylsalicylic acid in a continuous thin coating of ethylcellulose, developing microstructures for oral administration with modified drug release. The formulation released the drug substance for 4 h after administration, providing the patient with an 8 h analgesic effect. In addition to achieving prolonged release, the demonstrated technological approach also served as a strategy to reduce the irritating effect of acetylsalicylic acid on the gastric mucosa [10].

The properties of drug microcarriers depend mainly on the materials and methods used for their formulation. The material for building the microparticles must be able to provide the final product with specific characteristics and ensure the effective incorporation of the drug substance into it. High stability of the active substance, control over the drug release, and targeting of the therapeutic agent to the desired site in the body must be ensured. Many natural, semi-synthetic, and synthetic polymers can be used as drug carriers. Among them, natural polysaccharides such as starch, cellulose, and their derivatives have been widely used in the development of drug-delivery systems for oral, nasal, ophthalmic, and dermal applications due to their good tolerability, non-toxicity, mucoadhesive characteristics, good encapsulating properties, and low cost [11,12]. Biocompatibility and biodegradability are two of the most important characteristics of natural polymers as materials for drug-delivery systems. Biodegradability is of utmost importance to prevent acute or long-term toxicity [13]. Starch and its derivatives are defined as biodegradable polymers. This is because under physiological conditions, the chains in their molecules can be easily cleaved by various enzymes found in the mucous membranes and in the stomach or produced by the normal intestinal flora. Cellulose also does not accumulate in the body and is a non-toxic and safe material [14,15].

Starch and cellulose derivatives can be classified according to their structure, chemical composition, and sources. These natural polysaccharides can be obtained from plants, algae, lichens, and fungi, as well as some microorganisms by using different methods for extraction, purification, and separation. The resulting polysaccharides as a final product are characterized in terms of their molecular weight, monosaccharide composition, and total sugar content [16,17]. Polysaccharides usually have a high molecular weight and can establish multiple inter- and intra-molecular interactions due to their free hydroxyl groups. Thus, they can significantly increase the viscosity of the medium and cause its gelling [18]. These characteristics of starch and cellulose are essential for their particle-forming ability in order to formulate drug-loaded microstructures, as well as for the mechanism through which the polymer matrix/shell swells/degrades and releases the incorporated active substance. Controlled release of the included drug substances is an achievable and desirable feature of microparticle drug-delivery systems based on starch and cellulose polysaccharides. The drug-release rate from the microparticles is influenced mainly by the matrix structure, the polymer properties, and the drug substances [19]. Drug encapsulation in a slowly degrading matrix allows for delayed release, but this is not the only mechanism by which the incorporated substances are released from the microparticles. The process is usually diffusion-controlled. Drug molecules also pass through the pores formed in the process of particle formation. To obtain formulations with modified release, it is important that drugs are released prolonged at a constant rate over time. However, the release profile is often complex and involves an initial burst release of the drug from the peripheral parts of the particle, followed by a slower release due to diffusion and polymer degradation. Starch and cellulose derivatives can be easily modified through crosslinking processes or polyelectrolyte complexation, forming matrix structures providing systems with the desired delayed drug release [20,21,22,23].

The aim of this study is to review the main characteristics of natural polysaccharides: starch, cellulose, and their derivatives, which are related to their potential application as microparticulate drug carriers in the pharmaceutical field. To the best of our knowledge, there have been no reviews on this topic in the literature so far. By investigating the contemporary literature, we have highlighted the essential challenges in formulating polymer microparticles, proposed various technological solutions, and analyzed future perspectives for developing polysaccharide drug microcarriers.

## 2. Methods for Preparation of Polysaccharide Microparticles

**Figure 1:**
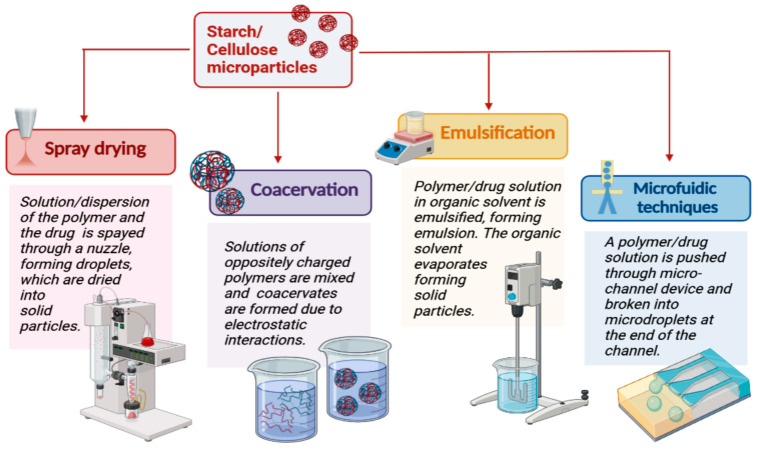
Different methods for the preparation of polysaccharide-based microparticles. Created with Biorender.com (accessed on 29 July 2023).

An important quality that makes starch and cellulose derivatives attractive drug microcarriers is their ability to easily form microparticles by classical microencapsulation methods. The most used techniques to produce polysaccharide drug-loaded microparticles are emulsification, spray-drying, and coacervation techniques, etc. (Figure 1).

The choice of microparticle-preparation technique depends on the properties of the drug substance and the desired application of the resulting microsystems. The emulsification techniques are a popular and reliable method for creating microspheres from starch and cellulose due to their simple technology and effectiveness. The polymer and the drug are dissolved in an organic solvent. The solution is emulsified in water, and microparticles are formed upon evaporation of the organic phase [24]. Typically, a water-in-oil emulsion (W/O) is created by mixing an aqueous solution of the polysaccharide and the drug substance with a suitable lipophilic phase, but other options are also possible, like preparing an oil-in-water (O/W) [25], a water-in-water (W/W) [26], or an oil-in-oil (O/O) [27] emulsion. The rate at which the emulsion is stirred is a crucial factor that affects the size and shape of the microparticles formed. An emulsifier is also included to stabilize the droplets of the dispersed phase [26]. The characteristics of polymer microparticles obtained by the emulsion technique are influenced by the composition of the emulsion (polymer concentration, type, and quantity of emulsifier), as well as the process parameters (stirring speed, duration, temperature, etc.). A higher concentration of polymer leads to an increase in the viscosity of the dispersed phase where it is added. This leads to the formation of larger droplets and microparticles. By increasing the stirring speed of the medium, the size of the resulting microstructures can be reduced. More energy is included in the system, which provides more efficient dispersion of the phase into the medium and the formation of finer droplets. The preparation of starch/cellulose derivatives microparticles by the emulsion technique with solvent evaporation is a longer process compared to other microencapsulation methods. Its duration depends on the evaporation rate of the solvent used. In most cases, heating of the system is required, which can affect the stability of the drug substances [24,25,26,27]. Various methods for forming microemulsions are described, which aim to optimize the process. Among them are emulsification using ultrasound or the use of high-pressure homogenizers, microfluidic, membrane emulsification, etc. [28].

Spray-drying is another effective method to develop microstructures from starch and cellulose derivatives. By changing process parameters, particle characteristics like size, distribution, shape, and morphology can be optimized. Spray-drying is a single-stage method of producing powders, transforming directly the starting liquid material into dry particles. The produced small droplets increase the surface area/volume ratio of the liquid, leading to rapid solvent evaporation. The sprayed droplets only have a few seconds to interact with hot gas to produce solid particles. However, this is not long enough to affect the stability of the substances used [29,30]. The concentration of the starting material for spray-drying, which includes the polymer and the drug substance, is a crucial technological parameter. It significantly impacts the characteristics of the resulting microparticles in spray-drying. Therefore, it is imperative to pay close attention to this parameter to ensure desirable outcomes. The speed of the peristaltic pump, the amount of compressed gas, the temperature, and the airflow rate are other crucial technological parameters that should also not be overlooked [31,32]. Freeze-drying is also a widely used drying technique for the formulation of starch and cellulose microparticles. The method allows obtaining polysaccharide microparticles with good physico-mechanical characteristics and stability [33].

Complex coacervation is another possibility for the formation of polysaccharide microsystems. In this technique, microspheres are formed through ionic interaction between solutions of oppositely charged polymers. Phase separation occurs in an aqueous medium due to the attraction between the opposite charges of the polymer molecules and the formation of polyelectrolyte complexes. The advantage of this method is the ability to use a wide range of natural and semisynthetic polymers, such as chitosan, alginate, carboxymethylcellulose, gelatin, and others [34,35]. A higher concentration of the polymer in the solution leads to larger microparticles and higher drug entrapment efficiency obtained through the phase-separation method. A low concentration of the polymer solution can result in microstructures with low density, wide size distribution, and rapid release of the encapsulated drug substance. A crucial factor in complex coacervation is achieving opposite charges between the two polymers. The ideal pH for effective phase separation varies based on the polymers used. The size and size distribution of microparticles depend on stirring speed and duration during coacervation. Excessive stirring may lead to smaller microspheres and reduced drug incorporation efficiency [8,36].

## 3. Starch-Based Microparticulate Drug-Delivery Systems

Starch is a polysaccharide of plant origin whose main function is to supply the plant with energy. It accumulates in the tissues and organs of almost all plants—in the roots, stems, shoots, leaves, fruits, and seeds. Cereals, potatoes, and corn are among the main sources of starch due to the high content of this polysaccharide [37].

### 3.1. Chemical Structure and Production of Starch

**Figure 2:**
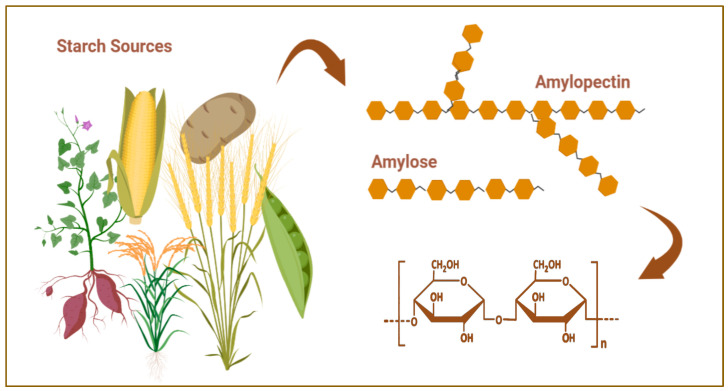
Starch sources and chemical structure. Created with Biorender.com (accessed on 29 July 2023).

The chemical structure of starch consists of two types of glucans: amylose and amylopectin. Unbranched amylose comprises glucose residues linked together by α-1,4-glycosidic bonds. Branched amylopectin has additional α-1,6-glycosidic linkages (Figure 2) [38].

The polymer chain of starch is stabilized by a large number of hydrogen bonds that form between its hydroxyl groups. Depending on its origin, the polysaccharide may show some structural differences, for example, the ratio of amylose to amylopectin and the shape and length of the polymer chains. Most commonly, amylose constitutes 16 to 35% of the polymer molecule [39]. Starch of natural origin does not swell and does not dissolve in cold water, but forms gels at high temperatures. Under the action of acids or enzymes, the length of the polymer chains is reduced, and they become soluble in water. Amylopectin and amylose are easily hydrolyzed by the enzyme α-amylase, which is secreted by the salivary glands and pancreas [40,41].

The industrial production of starch involves a series of stages, with the first stage aimed at separating the starch granules of the plant from its other components, such as proteins, cellulose shells, fibers, etc. This is achieved by physical methods, such as crushing, sieving, and centrifugation. The production scheme is individual for each raw material (maize, wheat, potatoes, etc.). In the second stage, starch can be subjected to direct drying, which leads to the so-called “native starch”, or undergo additional processing through various transformations, i.e., “modified starch”. In the third step, maltodextrins and polyols can be produced by hydrolysis reactions [42,43].

### 3.2. Preparation of Starch Microparticles

Interest in the development of starch microparticles as drug-delivery carriers dates back decades. A large number of studies in this field from recent years can also be found. This testifies to the many possibilities that these dosage forms offer and proves that there are still unresolved challenges in their development and implementation in practice. Starch microstructures are relatively easy to obtain by applying classical methods for the production of polymer microcarriers, such as emulsification techniques, spray-drying, coacervation, etc. Micro- and nanoparticles of different types of starch can be formed even by simple ultrasonic homogenization, without the addition of chemical reagents and without the need for further purification [44]. For example, starch suspensions with a concentration of 10% were sonicated at 25 °C and 20 KHz frequency for 30 min, then allowed to rest for 1 h. The formed coarse structures settled out (they underwent homogenization again), and the formed microparticles remained above them in the liquid phase. They were decanted and dried at 35 °C for 48 h. Microparticles were obtained using cassava starch, maize, and yam with a high yield of 88 ± 5% using this technique. From sweet potato starch, which contains the highest amount of amylose (30%), particles with the smallest dimensions of 1–3 μm were obtained, compared to those from cassava and corn, containing 18% and 25% amylose (3–7 μm, respectively). Besides microparticles, the authors also obtained starch nanostructures with the following dimensions: 8–32 nm (yam starch), 36–68 nm (corn starch), and 35–65 nm (cassava starch) [44].

Methods for preparing drug-loaded starch microstructures typically involve a crosslinking process of the polysaccharide during or after the preparation of the polymer particles. This is necessary because of the hydrophilic properties of starch to limit its dissolution under physiological conditions. Campos et al., for example, modified the starch structure by chemical crosslinking and used the resulting polymer to produce drug-loaded microparticles. The water-soluble polysaccharide first reacted with 2-vinyl-4,4-dimethyl-2-oxazolin-5-one, which is a donor of vinyl groups for the structure of starch. The polymer was then surface-crosslinked by dipropylene glycol diacrylate (DPGDA), and microparticles were formed by a W/O emulsion polymerization method. The resulting particles were spherical in shape and had an average diameter of 150 μm [45].

Zhu et al. used epichlorohydrin to crosslink polysaccharide and obtain Ca^2+^-loaded porous starch microparticles. An emulsion technique with subsequent alcohol-alkaline treatment was applied. The porous structure of the particles allowed easy water intake and rapid release of Ca^2+^ ions from the polymer. This accelerated blood clotting, stimulated platelet adhesion, and made the dosage form a promising product for surgical hemostasis [46].

Sodium trimetaphosphate (STMP), sodium tripolyphosphate (STPP), and phosphorus chloride (PCl_3_) can also be used as starch crosslinking agents. Sondari et al. prepared microparticles of starch as drug carriers using the emulsion technique and crosslinking the polysaccharide with STMP. FTIR analysis confirmed the successful modification of starch, proving the presence of phosphate groups in its structure. It was found that the use of emulsifiers with different values of hydrophilic–lipophilic balance (HLB) when emulsifying the starch solution—4.5, 5.0, 5.5, and 6.0—had an influence on the diameter of the polymer structures. At the highest HLB, when co-surfactants were included, the microparticle size of the crosslinked starch was reduced [47].

STPP was used by Obireddy et al. as a crosslinking agent in the simultaneous incorporation of ketoprofen and ofloxacin into starch microparticles. They used a hydroxyethylated polysaccharide that persisted longer in the circulation and was characterized by controlled degradation in the presence of serum α-amylase. The drug-loaded microparticles were obtained by ultrasonic homogenization and showed drug entrapment efficiency (EE) between 40.2 and 54.4%. The amount of crosslinking agent used during particle formation was found to have a negative impact on EE. That was explained by the formation of a denser polymer matrix as a result of crosslinking, which had a smaller free volume for encapsulation of the two drug substances [48].

Another option for modifying the structure of starch is the addition of acrylic groups to the polysaccharide chain. Microparticles of polyacrylic starch can be obtained from hydrolyzed starch (molecular weight 5000 Da), which reacts with glycidyl acetate. The polymer obtained is thus subjected to W/O emulsion polymerization to form microparticles. The degree of modification of the polysaccharide is determined by the number of acrylic groups per glucose residue. The porosity of the resulting particles depends on it, which affects the stability of the formulation in vivo. With this preparation method, about 90% of the particles have a diameter of <3 μm, their average size being about 2 μm. Protein antigens with free amino groups can be conjugated to the resulting polymer microstructures using carbonyldiimidazole (CDI). It was found that protein binding did not result in significant changes in particle size. The hydrocarbon bonds slowed down the enzymatic degradation of the polymer in saliva, gastric juice, and intestines. Moreover, human-serum-albumin-conjugated polyacrylic starch microparticles were reported to be more effective than the unbound therapeutic agent due to the adjuvant effect of the polysaccharide [49].

Protein antigens can be delivered by a starch carrier even without covalent binding to the polymer, but only by physical incorporation into the polymer matrix. For example, starch microparticles were formulated by Heritage et al. using a simple emulsion technique. Hydrolyzed starch was dissolved in dimethylsulphoxide at a high temperature, and the solution was subsequently cooled down. The protein solution was added to the polysaccharide and the resulting mixture was emulsified in the oily phase under continuous stirring. Microparticles were formed by dripping the emulsion into an acetone medium containing surfactant (e.g., Tween 80). They were separated by filtration, typically having sizes between 1 and 100 μm, a mean diameter of 4–5 μm and an antigen content of 5–6% [50].

Drug loading of starch microparticles can be carried out both during preparation and after the particles are formulated—the final polymer structures can absorb the active substances into their matrix. Choi et al. developed starch microparticles by freeze-drying. They used the resulting porous structures as carriers of resveratrol—a lipophilic polyphenol compound that can be easily degraded by heat or light. The polysaccharide microparticles were dispersed in a 30% *w*/*v* solution of the active substance in ethanol and polyethylene glycol 400 (ratio 40:60 *v*/*v*), and resveratrol was diffused into the polymer matrix. After ethanol evaporation, drug loading of 112 mg resveratrol was determined for 1 g particles, which retained more than 92% after 110 days of storage. The proposed formulation demonstrated 32% higher stability of resveratrol to UV radiation and 25% greater iron-reducing activity compared with the drug substance [51].

Besides being drug carriers, starch microparticles have also been used as adjuvants for anti-tumor therapy. The Swedish company Pharmacia AB has been developing degradable starch microspheres (DSMs) called Spherex^®^ [52]. They were used as an arterial embolizing agent in chemotherapy and were the first such product to be authorized in Japan. DSMs consist of spherical particles of about 45 μm diameter obtained by emulsion polymerization of partially hydrolyzed potato starch using epichlorohydrin as a crosslinking agent. They are characterized by a gradual degradation by amylase in the blood and have a 20–35 min in vitro half-life. It was clinically established that a temporary embolization was observed after arterial administration of Spherex^®^ in arterioles. In addition, when introducing the microspheres into the hepatic artery, along with an anti-cancer agent, a slowdown in blood flow occurred, which allowed longer maintenance of a high drug concentration in the tumor area. As a result, the local effect of the therapeutic agent was enhanced, and its systemic side effects were limited. Clinical studies involving the administration of DSM in combination with mitomycin C in metastatic liver tumors showed twice the therapeutic efficacy (54.5%) compared to arterial injection of the drug without the starch microparticles (20.0%). Similar results have been described using Spherex^®^ in the therapy of liver metastases from colorectal tumors, as well as in combination with doxorubicin and cisplatin [53,54].

### 3.3. Preparation of Maltodextrin Microparticles

Dextrin is formed when processing starch with an acid or a base under heating, which results in the formation of highly branched polymer chains. Different products can be obtained depending on the conditions applied. Compared to unmodified starch, dextrins have improved water solubility and form solutions with lower viscosity [55]. Maltodextrin (MD) is produced by acidic and/or controlled enzymatic hydrolysis of starch and it is composed of D-glucose blocks bound by α-(1,4) and α-(1,6) bonds. It contains 2–3% glucose and 5–7% maltose. It is easily soluble in water and slightly or almost insoluble in alcohol [56]. The extent of starch degradation in the obtained maltodextrin is referred to as dextrose equivalent (DE). DE is inversely proportional to the degree of polymerization (DP) of the dehydrated glucose units and estimates the content of the reducing end groups in the polymer structure [57]. MD has a DE between 3 and 20, indicating that its carbohydrate chain is long and represents a complex mixture of high- and low-molecular-weight components. As a starch hydrolysis product, maltodextrin has, in part, the structure of amylose and amylopectin [58]. Polymers with different DE values exhibit different chemical and physical properties, such as solubility, freezing temperature, viscosity of solutions, etc. [59]. It has been reported that with an increase in the DE of the polysaccharide (and consequently an increase in the degree of hydrolysis), its molecular weight and the degree of linearity of its chains are lowered [60]. For a microencapsulation process, the use of maltodextrin with higher DE values is preferred because such polymers lead to the preparation of particles with a smoother surface, fewer cracks, and therefore higher encapsulation efficiency and increased stability of the encapsulated material [61]. On the other hand, Zhu et al. found that by increasing DE, the hygroscopicity of the obtained material by spray-drying polysaccharide microparticles is increased. This is explained by the more branched structure of maltodextrin and the presence of more free hydrophilic groups that interact with moisture from the air [62].

MD is widely used for the microencapsulation of biologically active substances due to its high water solubility, low viscosity at high concentrations, low cost, neutral taste, and aroma [63]. In addition, maltodextrin is a preferred coating material that is characterized by high thermal and acidic stability [64,65]. At the same time, it can provide protection from oxidation of the medicinal substances encapsulated inside [66]. MD has been commonly used in the preparation of microparticles by spray-drying. It is usually added to the sprayed emulsion, helping the formed droplets to harden more efficiently and form a crust around them during the spray-drying process [40]. The use of maltodextrin during drying and storage affects hygroscopicity and glass transition temperature and increases microencapsulation efficiency [67]. Due to its low emulsifying ability, MD is usually mixed with other encapsulating polymers, such as Arabic gum, modified starch or proteins like whey, casein, inulin, etc. [58,68,69]. The combination of maltodextrin with some proteins has been reported to induce a Maillard reaction that produced conjugates with good emulsifying properties and, accordingly, more stable emulsions from which microparticles can [70,71]. For example, authors obtained microstructures of MD and casein, observing hydrogen bond formation and a crosslinking reaction when mixing the polymers. As a result, the secondary structure of casein changed, which favored the emulsification process [72,73]. Shao et al. reported that adding protein to maltodextrin led to a significant increase in the effectiveness of drug incorporation in the formulated polymer microparticles [74].

Among the most used methods for the preparation of microparticles of maltodextrin are complex coacervation [75,76], spray-drying [77,78,79,80], freeze-drying [64,81], an emulsion technique [82], and a combination of these methods, e.g., complex coacervation and spray-drying [83,84,85,86]. New microencapsulation approaches are also being developed, such as the Pickering emulsion technique [87,88]. In this method, a thermodynamically stable emulsion is formed without the use of an emulsifier by adsorbing a solid phase at the water/oil interphase. The liquid from inside the particles can be removed by freeze-drying or heat-induced evaporation, during which the oil droplets are embedded in the matrix of solid particles [89].

### 3.4. Preparation of Cyclodextrin Microparticles

**Figure 3:**
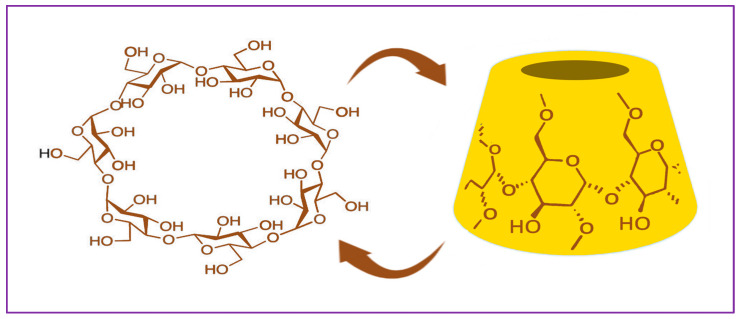
β-Cyclodextrin chemical structure. Created with Biorender.com (accessed on 29 July 2023).

Cyclodextrins (CD) are starch derivative polymers composed of α-D-glucopyranose molecules bound together in an annular form. They are cyclic oligosaccharides, which are also called cycloamyloses, cyclomaltases or Schardinger’s dextrins. Depending on the number of glucose units in their structure, CDs are referred to as α-cyclodextrin (six glucose units), β-cyclodextrin (seven glucose units), and γ-cyclodextrin (eight glucose units) [90]. The glucopyranous structures in CDs are linked by α-1,4 bonds and give the cyclodextrins a typical conical shape characterized by an internal hydrophobic and an external hydrophilic surface (Figure 3). On the upper surface of the formed cyclic structure, hydrogen bonds between 2-OH and 3-OH groups are observed, which are weaker in α-CD and stronger in γ-CD. Around the lower part of the cone, 6-OH groups can also form hydrogen bonds, but they are easily destabilized under the influence of dipolar effects and are rarely preserved in cyclodextrin crystals [91]. The hydrophobic cavity that cyclodextrins form allows the incorporation of lipophilic drug substances into the micro- and nano-structures of CDs. Free hydroxyl groups enable cyclodextrins to bind to other polymers and form polymer complexes. Also, these groups can be oxidized, esterified, or crosslinked, allowing the preparation of various cyclodextrin-derived materials [92].

Cyclodextrins were discovered in 1891 by Villiers, who described a new crystalline substance produced by the degradation of starch by *Bacillus amylobacter*. These cyclic polysaccharides were found to originate from enzymatic processing of starch under the action of glycosyltransferase CGTase and α-amylases. The starch was first liquefied at a high temperature or by the addition of α-amylase. Furthermore, due to CGTase, all types of CDs can be synthesized in ratios depending on the specific type of enzyme used. The three types of cyclodextrins can be easily purified on the basis of their different solubility. Β-CD can be isolated by crystallization since it has very low solubility in water. The other two forms are isolated by chromatographic methods or by the addition of complexing agents, such as toluene and ethanol [91].

The three CD formations have a similar structure. They are crystalline and non-hygroscopic in nature. The diameter of their cavities is different and depends on the number of glucose units. α-CD forms a small cavity, which determines the more limited application of the polymer. β-form is the easiest to isolate and is the most economically advantageous. Its structure is characterized by a moderately large cavity, and it is the most widely used form of cyclodextrin. γ-CDs possess the largest cavity, but they are not subject to extensive research and their application as drug carriers has not been well studied [93]. β-CD has less water solubility than α-CD, although it contains a greater number of hydroxyl groups, which is due to the internal network of hydrogen bonds between secondary hydroxyl groups. Some of the main characteristics of the three forms of cyclodextrin are described by Loftsson et al. and are summarized in Table 1 [94].

As a safe and affordable natural material, cyclodextrins are widely used in various fields of medicine and pharmacy, including as polymer carriers of drugs, especially suitable for the microencapsulation of substances with hydrophobic properties, for example, essential oils. Various methods for the preparation of microcapsules from CD have been described, and among them the most popular are as follows: inclusion complexation, kneading or paste method, recrystallization, or co-precipitation and the ultrasonic method. To obtain the final powder product, the resulting emulsion or suspension is most often subjected to drying using different approaches, such as freeze-drying or spray-drying.

Incorporation by complexation is a physical method for the preparation of microcapsules with CD, which is based on the structural characteristics of cyclodextrin. The hydrophobic internal and hydrophilic external conical formation of the polysaccharide allows its selective binding to lipophilic molecules in aqueous solution through van der Waals forces, hydrogen bonds, dipole–dipole interactions, and microcapsule formations of varying stability [95].

In the kneading method, the active substance is added to a paste of cyclodextrin containing 10–40% water. The advantage of this method is that no additional solvent is used and therefore less energy is spent on drying [96]. The CD is mixed with a small amount of deionized water and homogenized until a paste is formed, after which the drug substance is added. The resulting composite can be directly dried or washed with a small amount of water and then separated by filtration. Depending on the properties of the encapsulated substance and the amount of water used, the paste may dry to form a coarse solid material rather than microparticles. It should be dried well and ground to a fine powder. A disadvantage of this method is the limited effectiveness of drug incorporation in the final product [97].

Recrystallization, or co-precipitation, is the most commonly used laboratory technique to obtain CD microcrystals. It involves preparing a saturated solution of cyclodextrin at a high temperature to which the drug substance for microencapsulation is added. The temperature is gradually lowered, changing the solubility of the CD, the polymer crystallizes, and the resulting material is separated by centrifugation or filtration and dried to obtain particles [98]. By this method, for example, microcapsules of β- and γ-CD were obtained, including the essential oil of *Lippia graveolens* [99]. Yang et al. used the co-precipitation technique to encapsulate estragole in β-CD, aiming to improve its thermal stability and achieve controlled release. The solubility of estragole increased proportionally with increasing β-CD concentration, which was explained by its interaction with the polysaccharide [100].

Another approach to obtain drug-loaded cyclodextrin microparticles involves the use of ultrasound. A solution of CD and the drug substance is prepared and sonicated at a certain intensity. This facilitates the incorporation of the drug molecule into the cyclic polymer cavity. This is usually performed at high temperatures, and after cooling, the resulting precipitate is separated, washed, and dried. The whole process is relatively fast, easy, and convenient, even for the industrial production of cyclodextrin microcapsules [91]. Shi et al., for example, prepared β-CD microcapsules with cinnamon oil using sonication. They reported a 38% encapsulation efficiency of the essential oil in the particles, achieving increased oil stability and proving that its main components did not change significantly after incorporation into the polymer [101].

Other recent examples of microparticles formulated from cyclodextrin and other starch derivatives are presented in Table 2.

## 4. Cellulose-Based Microparticulate Drug-Delivery Systems

Cellulose is one of the most abundant biopolymers on Earth. It is the main structural component of the cell walls of lower and higher plants. Cellulose is synthesized by many organisms, including bacteria, algae, fungi, and different tree species. Cotton fibers are the purest form of plant cellulose, containing up to 90% cellulose, while the polysaccharide content of woody biomass ranges between 40 and 50% [131].

### 4.1. Chemical Structure and Production of Cellulose

**Figure 4:**
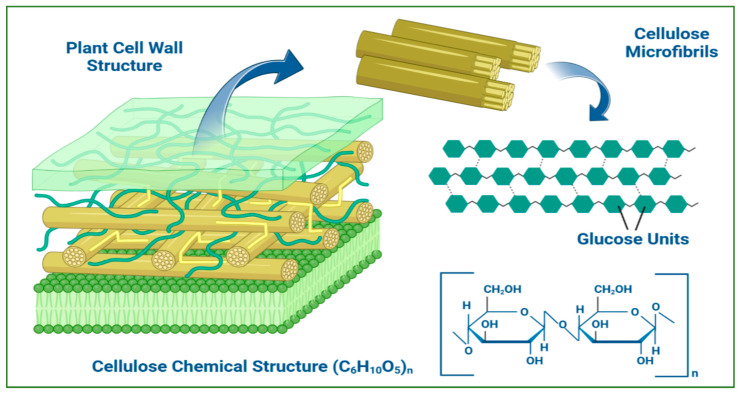
Cellulose structure and function in plant cells. Created with Biorender.com (accessed on 29 July 2023).

Cellulose is a homopolymer of glucose like starch, but its glucose monomers are bound by β-1,4 bonds (Figure 4). Depending on the number of monomers, its molecular weight can reach over 100,000 Da. The cellulose chains are unbranched and arranged parallel to each other. They are connected to each other by H-bridges formed between hydrogen atoms and hydroxyl groups of glucose monomers. Thus, cellulose chains form the so-called microfibrils, which in turn are grouped into larger structures (bundles of microfibrils). These fibrils build the wall of the plant cell, and their arrangement promotes the stability of plant parts and determines the good mechanical properties of cellulose: a robust, fibrous, and water-insoluble polysaccharide [132,133].

Cellulose is mainly used for paper production and in the textile industry (cotton, flax, and other natural fibers). In recent years, cellulose derivatives have found wider applications, especially in pharmaceutical practice. Cellulose can be transformed by various modifications, such as etherification (cellulose ethers), esterification of its hydroxyl groups (cellulose esters), or depolymerization [134].

Cellulose and its derivatives, such as cellulose ethers, esters, and oxycellulose, are widely used in the development of various drug-delivery systems. The largest group among them are polysaccharide ethers, which include five main derivatives: ethylcellulose (EC), methylcellulose (MC), sodium carboxymethyl cellulose (CMC), hydroxypropyl cellulose (HPC), and hydroxypropyl methylcellulose (HPMC). Cellulose ethers are used as important excipients in pharmaceutical practice for the design of matrix and reservoir drug systems. After administration in the body, they swell in water and form a hydrogel layer that begins to grow around the drug core. The hydrogel layer constitutes a diffusion barrier while allowing the penetration of water molecules into the polymer matrix to release the drug substance [135]. Cellulose ethers are obtained by alkaline treatment of the polysaccharide with suitable reagents. The properties of the derivatives obtained, such as solubility, viscosity, and surface activity, depend essentially on their chemical structure and the distribution of their functional groups. With the increase in the degree of substitution, ethers gradually pass from water-soluble to soluble only in organic solvent molecules [136]. The solubility of cellulose ethers is different, which determines differences in the way they release the drug substances included in their matrix.

Ethylcellulose is a nonionic cellulose ether that is not affected by pH. It is insoluble in water but can be dissolved in a few organic solvents [137]. For drug-delivery systems, EC can be used alone as a carrier or in combination with water-soluble polymers. The polysaccharide commonly serves as a coating that ensures delayed drug release from the matrix.

Methylcellulose can be dissolved in water, giving clear to slightly opalescent viscous solutions [138]. By increasing the degree of the polymer substitution, the solubility of the polysaccharide decreases due to the blockage of polar hydroxyl groups in its structure. Methylcellulose solutions are stable over a wide pH range from 2 to 12 without any visible changes in their viscosity. The polymer has been used as a carrier primarily of water-soluble drug substances, aiming at increasing their solubility and bioavailability [139]. Carboxymethyl cellulose is a polyanionic, water-soluble cellulose derivative that is produced through a carboxymethylation process [140].

Hydroxypropyl cellulose is obtained using propylene oxide, which reacts with the anhydrous glucose chain of alkaline cellulose. HPC is soluble in a range of organic solvents and even in cold water [141]. Hydroxypropyl methylcellulose has been applied to produce modified-release formulations since the early 1960s [142]. It has a hydrophilic polymer structure, and it is water-soluble. After administration of an HPMC-encapsulated drug substance, water gradually penetrates and hydrates the polymer chains, which leads to the release of the active components from it. The drug-release process is related to two main mechanisms: diffusion through the formed gel layer of the polymer and erosion of the matrix [143]. In the case of a water-soluble drug substance, its release from the HPMC carrier will depend primarily on the diffusion process. However, if the drug substance is slightly soluble/insoluble in water, or if the polysaccharide is with low molecular weight, the predominant release mechanism is erosion of the polymer matrix [144].

### 4.2. Preparation of Cellulose Microstructures

The preparation of cellulose products is a subject of interest to scientists working in the fields of chemistry, chemical engineering, biochemistry, and many other areas related to the design of biological materials. This polysaccharide is a widespread and renewable bioresource that possesses excellent mechanical and chemical properties. Moreover, cellulose can be easily modified chemically, allowing adjustment of its properties to the desired application. The polymer can be given hydrophilic or hydrophobic characteristics. It may be loaded with either anionic or cationic charge [132].

Widely used cellulose microparticles are the so-called cellulose beads, which are usually more than 10 μm in diameter. They are used in many areas, including protein wrapping, targeted drug delivery, formulations of modified-release drug systems, etc. In recent decades, various methods for the preparation of cellulose microparticles have been described, involving the use of various solvents, techniques for the precise shaping of spherical structures, as well as technological devices for industrial production. A number of functionalized materials based on cellulose have been developed for specific applications by introducing additional chemical groups to its structure or mixing the polymer with other organic and inorganic compounds. Cellulose microspheres are even commercially available as pharmaceutical products with certain properties and size [145].

The preparation of spherical cellulose particles was first described in 1951. The resulting structures with an average diameter of 2 mm, then called pellets, were formed by simple dropping of a viscous cellulose solution in an aqueous medium containing a gelling agent [146]. After this development, various techniques for obtaining polysaccharide particles of a significantly smaller size in the microfield were studied. For the formation of microparticles from cellulose/cellulose derivatives, methods such as emulsion technique, spray-drying, and freeze-drying are most often applied. The principle of obtaining microspheres from the polysaccharide includes three main stages: dissolving cellulose (or cellulose derivatives), shaping microdrops from the polysaccharide solution, switching from sol to gel, and solidifying the drops in solid particles. In addition, pre- or post-processing is often carried out, which aims to provide the final product with the desired characteristics.

**Figure 5:**
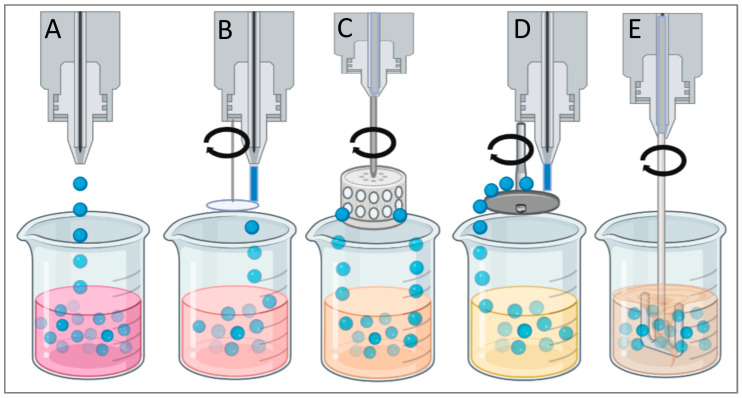
Schematic illustration of different techniques for preparing cellulose microspheres: (**A**) dripping, (**B**) jet cutting, (**C**) rotational drop formation, (**D**) rotating disc, (**E**) dispersing. Created with Biorender.com (accessed on 29 July 2023).

Although different derivatives of the polysaccharide and different solvents can be used for the preparation of cellulose microparticles, in all techniques, the shaping of microspheres from the polymer solution is achieved either by dropping or by a dispersion method (Figure 5) [145].

In general, the diameter of the cellulose microparticles obtained by the dropping techniques is over 500 μm and depends on the size of the droplets that are generated. The formation of smaller structures can be achieved by using vibrating nozzles, air jets, or cutting discs (Figure 5B) directed to the flow of the polymer solution [147,148]. An efficient technique allowing the generation of a large number of microdroplets within a short time involves the use of a rotating cylindrical vessel (cup) with fine holes through which the cellulose solution passes (rotational formation of drops, Figure 5C) [149]. By varying the speed of rotations, the geometry of the vessel, and the size of its openings, the size of the resulting microparticles can be controlled. Another possible method for the formation of microdroplets from cellulose is the use of a rotating disc (Figure 5D). A thin film of the polymer solution is spread at a constant speed on a rotating disc, resulting in centrifugal forces, forming droplets that are ejected from the edge of the disc [150].

Cellulose particles, substantially smaller than 500 μm, are obtained by dispersion techniques. A solution of the polysaccharide is dispersed in an immiscible liquid phase with opposite polarity at a high stirring rate. This leads to the formation of an emulsion that can be stabilized using surfactants. The resulting system contains droplets of the dissolved polymer, which can be dried into solid microparticles with a diameter of between 10 and 100 μm. The diameter of the cellulose structures formed by this technique is determined by the dispersing rate, the type and number of emulsifiers used, the ratio of hydrophobic to hydrophilic solvent, and the viscosity of the dispersed medium and the cellulose solution [151]. Examples of microspheres from cellulose and its derivatives are presented in Table 3.

### 4.3. Formulation of Cellulose Drug-loaded Microparticles

Rama et al. developed zidovudine-loaded ethylcellulose microparticles as controlled-release drug systems. An emulsion technique (double water–oil–oil emulsion) with solvent evaporation was applied. A mixture of acetonitrile and dichloromethane was used in a 1:1 ratio and liquid paraffin as primary and secondary oil phases, respectively. The resulting microparticles had a spherical shape, 41–55% drug load, and delayed release of zidovudine for 18–20 h in phosphate buffer with pH 7.4, which corresponds to Higuchi’s kinetic model [163]. The same method for preparation was applied for incorporating diclofenac into ethylcellulose microspheres. The prepared structures had a drug loading of 51% and released the incorporated diclofenac in vitro for 12 h [164]. Analogically, microspheres with sustained release of salbutamol up to 10 h were formulated by Nath et al. [165].

Drug-delivery microsystems with ethylcellulose can also be formed by an O/W emulsion technique with solvent evaporation. Through this method, the use of 2^3^ full factorial design, acyclovir-loaded ethylcellulose microparticles was developed by Cheu et al. [166]. The influence of the polymer solution viscosity, polymer/drug substance ratio, and polysaccharide concentration on encapsulation efficiency and release profiles was studied. At a higher viscosity and larger CH_2_Cl_2_/ethylcellulose ratio, an increase in the efficiency of acyclovir incorporation into microparticles was observed. The proposed structures were characterized by a delayed in vitro drug release (more than 12 h), which was pH-dependent. The dissolution rate was greater in an alkaline medium compared to a medium mimicking gastric liquid. In another study, a Box–Behnken experimental model with three factors and three levels was applied to determine the influence of the main technological parameters for obtaining drug-loaded microparticles of ethylcellulose by an emulsion technique. The microparticle models had a yield of 42.29–97.22% and a drug load of 2.18–24.55% [167].

Another oral antidiabetic agent, glipizide, was included in a microcarrier, composed of two cellulose derivatives. A solution of ethylcellulose in chloroform containing the drug substance was emulsified in the form of fine droplets in an aqueous solution of sodium carboxymethyl cellulose. As a result of intensive stirring at room temperature for 3 h, chloroform evaporates and polymer microparticles were formed. The resulting drug systems had an average diameter of 300–600 μm, 81–91% drug entrapment efficiency, and in vitro drug release over 10 days, and the observed hypoglycemic effect after their administration to rabbits lasted up to 6 days [168].

Wasay et al. used hydroxypropyl methylcellulose to produce microparticles loaded with meloxicam. An emulsion technique with solvent evaporation was applied, with the formed structures ranging between 90 and 150 μm in size. By incorporating the drug substance into microparticles from HPMC, the authors achieved modified drug release and significantly increased drug bioavailability determined after application on rabbits [169]. In another study, hydroxypropyl methylcellulose was used to produce curcumin-loaded delivery systems. Microparticles were formed by the spray-drying method from a water suspension of the polymer, lactose, and the drug substance under the following conditions: inlet temperature, 140 °C; outlet temperature, 125 °C; peristaltic pump speed, 15 rpm/min; gas pressure, 0.65 MPa; and nozzle diameter, 1.0 mm [170]. Javed et al. optimized the release of nifedipine from microparticles obtained from HPMC and polycaprolactone by an emulsion technique with solvent evaporation. The developed models showed a controlled release in both acidic and alkaline pH, which was affected by the ratio between the two polymers [171]. A coacervation technique was applied to form microparticles of hydroxypropyl methylcellulose phthalate loaded with ibuprofen. For phase separation, a 20% sodium sulphate solution was added to the polymer solution [172].

Singh et al. designed hybrid microcapsules of carboxymethyl cellulose and chitosan for microcapsulation of probiotic bacteria. Polymer microstructures were obtained by injecting a solution of both polymers through a nozzle and subsequent crosslinking with genipine. The microcapsules size was 5–10 μm. They showed high stability in an acidic medium, and at pH 7.4, they swelled intensively, which made them suitable carriers for targeted delivery to the intestine [140]. Similar micro-sized polyelectrolyte complexes between chitosan and carboxymethyl cellulose have also been described by other authors [173]. The size of the resulting structures was found to be influenced to a great extent by the ratio between the two polymers. The microparticles had an amorphous structure and the potential for microencapsulation and controlled release of thermosensitive biologically active substances, namely vitamins, antioxidants, phytosterols, and probiotics.

Other recent examples of microparticles formulated from cellulose derivatives are presented in Table 4.

**Table 4:** Studies on cellulose-derivative-based microparticles in the period 2019–2023.

Polymer	Preparation Method	Active Substance	Reported Results	Reference
Ethylcellulose	Spray-drying	Rupatadine fumarate	Mean diameter 1.2–4.9 µm; drug encapsulation 42–99%; taste masking of bitter organoleptic properties in vivo in human taste panel.	[174]
Sodium carboxymethyl cellulose	Spray-drying	Sildenafil citrate	Mean diameter 2–5 µm; aerodynamic properties for pulmonary delivery; Low toxicity on cell viability; higher lung/blood C_max_, AUC, extended half-life	[175]
Ethylcellulose	Spray-drying	Pirfenidone	Spherical particles with smooth surface; average size of 4 µm; sustained drug release intended for inhalation and targeted delivery to the lungs.	[176]
Hydroxypropyl methylcellulose	Spray-drying	Levodopa, carbidopa	Drug loading of 19.1%; encapsulation efficiency of 95.7%; sustained in vitro release 80% released drug after 12 h; high blood concentrations in vivo in rats.	[177]
Cellulose nanocrystals	Emulsification	Curcumin	Core-shell microparticles formulation using interparticle interactions; mean diameter 1–4 µm; increased drug retention in the particles (76.41%).	[178]
Hydroxypropyl methylcellulose	Emulsification	Curcumin	Mean microparticles diameter 30.2–76.7 μm; encapsulation efficiency 78.8–96.2%; sustained drug release, significant anti-arthritic, and anti-inflammatory activity.	[179]
Carboxymethylated diethylaminoethyl cellulose	Ionotropic gelation	Drug-free carrier	Microcarriers produced by ionic crosslinking of carboxymethylated soluble multifunctional cellulose; support cellular attachment and proliferation; promising materials for cell therapy and tissue engineering applications.	[180]
Carboxymethyl cellulose	Extrusion, ionotropic gelation	Esculin	Spherical shape particles; diverse morphology; physically stable in various media; controlled drug delivery with pH-triggered drug release;	[181]
Ethylcellulose	Emulsification	Metformin	production using oil-in-oil solvent evaporation technique; spherical microspheres with high drug entrapment efficiency and sustained drug release.	[27]
Ethylcellulose, casein	Freeze-drying	Curcumin	Entrapment efficiency 92.4%; average size 1.47 μm; prolonged drug release; antibacterial activity, i.e., loss of membrane integrity due to ROS production.	[182]
Ethylcellulose, polyethylene glycol	Emulsification	Metformin	Drug encapsulation efficiency 33–82%; particle size 15–178 μm; sustained drug release at pH 6.8–91% drug released in 12 h with Fickian diffusion mechanism.	[25]
HPC, HEC and CMC	Ionotropic gelation	Bovine serum albumin	High protein encapsulation efficiency; sustained drug release in acidic medium and high cumulative release in simulated intestinal fluid medium (86.17%).	[183]
Bacterial cellulose, collagen	Inverse suspension regeneration	Bovine serum albumin	Porous microspheres beneficial to the proliferation of MC3T3 E1-cells; drug-release kinetics described by the first-order release model.	[184]
Ethylcellulose	Coacervation	Glipizide	Microparticle yield 76%; particle size 50–450 µm; entrapment efficiency 89.8%; 50% reduction in plasma glucose level compared to conventional dosage forms.	[185]
Ethylcellulose	Emulsification	Nifedipine	Production yield 56–94%; average particle size 223–446 µm; initial burst release, followed by sustained release up to 12 h.	[186]

HPC: hydroxypropyl cellulose; HEC: hydroxyethyl cellulose; CMC: carboxymethyl cellulose.

## 5. Challenges and Future Perspectives for Polysaccharide Microparticle Formulation

The development of drug microcarriers based on polysaccharides starch, cellulose, and their derivatives has been ongoing for years and still continues to develop. Proof of this is the numerous studies that have been discussed so far, as well as the various patents recently published (Table 5). However, this area of pharmaceutical technology is facing some unresolved issues. One of the main challenges in this field is not only to develop a polysaccharide microparticle formulation, but to authorize it as a safe and effective therapeutic product for use on the pharmaceutical market.

For industrial production of polysaccharide microparticles, the formulation process is required to ensure high reproducibility. Most of the widely used methods for obtaining microparticles from starch and cellulose cannot provide this. Typically, in the batches obtained, the microparticle size varies in a wide range, making it difficult to characterize the developed product accurately and predict its pharmacokinetic behavior. In this direction, new approaches are being developed to obtain monodisperse microparticles with high reproducibility [187]. Most of these techniques are still not sufficiently studied and are not adapted to serial industrial production.

Another difficulty in developing microparticles as powder formulations is the great cohesiveness of micro-sized structures, due to their small size and large free surface, and their tendency to aggregate. This determines unsatisfactory rheological properties, which may be a prerequisite for inaccurate dosing of the powder dosage form [188,189]. The search for suitable excipients and approaches to improve the flowability of microparticles is mandatory if they are to be administered in the form of powders.

In order to be released on the pharmaceutical market, any medicinal product is subject to strict safety regulations, which include not only in vitro and in vivo animal studies, but also the mandatory conduct of clinical trials. There are clinical data in the literature on the application of the discussed polysaccharides, but there is a lack of data on such trials with drug microstructures based on them. Starch, dextrins, and cellulose derivatives are allowed for biomedical applications, but their use as pharmaceuticals in clinical trials is limited due to regulatory issues related to their source and characterization. Purity is critical for natural polysaccharides, which can have impurities, such as high bioburden, bacterial, and protein contamination, making total protein content estimation a significant concern. To ensure pharmaceutical product polymer quality and safety, manufacturing guidelines for pharmaceutical grade polysaccharides should be established [36,190].

**Table 5:** Patents related to the formulation and application of microparticles developed from starch, cellulose or their derivatives.

Patent Code	Patent Content	Year	Reference
WO2023145417A1	An adjuvant and vaccine composition containing an adjuvant and a complex including microparticles of a biodegradable polymer and cyclodextrin.	2023	[191]
CN115678222A	Biodegradable polymeric microparticles consisting of cellulose nanoparticles or hydroxyapatite nanoparticles and methods of making and using them.	2023	[192]
US20220315671A1	Product of crystalline starch nano-microparticles, procedures and gel for various applications.	2022	[193]
GB2615103A	A method of forming a composition comprising a probiotic microencapsulated in a denatured plant protein and maltodextrin matrix.	2022	[194]
WO2022129661A1	Amphoteracin B formulations for inhalation based on collapsed microparticles containing carbohydrates γ-cyclodextrin and mannose.	2022	[195]
WO2023286049A1	Protein containing bio-active compositions comprising cellulose microparticle carriers	2022	[196]
US20220213298A1	Porous cellulose microparticles and methods of manufacture thereof.	2022	[197]
US20220313617A1	Biotherapy for viral infections using biopolymer-based micro/nanogels based on chitosan and hydroxyethyl cellulose.	2022	[198]
AU2021103734A4	A method for synthesizing repaglinide-loaded floating microparticles based on ethyl cellulose and sodium alginate.	2021	[199]
WO2022200636A1	Pharmaceutical formulations and methods for the production of microparticles comprising oil, water, inulin fiber, and maltodextrin or cyclodextrin.	2021	[200]
CN110613700A	Omeprazole microparticle sustained-release pharmaceutical composition using starch and preparation method thereof.	2019	[201]
MX2016005434A	Crude starch microparticles as an adjuvant in vaccines, generating protective immune response against Mycobacterium tuberculosis infection.	2016	[202]
CN106039389A	Starch porous microparticle for hemostasis with a wide application prospect and stable preparation technology.	2016	[203]
EA201600005A1	Anti-tumor formulation based on recombinant Interferon alpha-2b in the form of microparticles for parenteral administration.	2015	[204]

## 6. Conclusions

Drug delivery through polymer microcarriers has been the focus of many studies, and still this pharmaceutical area is constantly evolving at high rates. The wide variety of synthesized polymers in recent years provide an opportunity to design drug-delivery systems with precisely determined physicochemical and biopharmaceutical characteristics. However, the risk of toxicity of novel and not thoroughly investigated materials remains a great limitation for the safe application of their therapeutic formulations. Natural polysaccharides, such as starch, cellulose, and their derivatives, have been extensively analyzed and tested in vitro and in vivo over the years and have been determined as harmless and tolerable drug carriers. The reviewed developments related to designing drug-loaded microparticles using starch and cellulose indicate the huge potential of these polymers for creating novel effective and safe drug formulations. Despite the large amount of data accumulated so far, additional, even more in-depth studies would make the most of the numerous advantages of these drug systems, as well as create new opportunities for improved drug delivery.

## Figures and Tables

**Table 1:** Physico-chemical properties of the three forms of cyclodextrins.

Properties	Cyclodextrin Form		
	α-CD	β-CD	γ-CD
Number of glucopyranose units	6	7	8
Molecular mass of anhydrous form (Da)	972.84	1134.98	1297.12
Solubility in water at 25 °C (mg/mL)	129.5 ± 0.7	18.4 ± 0.2	249 ± 0.2
Cone height (nm)	0.78	0.78	0.78
Inner diameter of the cone (nm)	0.50	0.62	0.80
Outer diameter of the cone (nm)	1.46	1.54	1.75

**Table 2:** Studies on starch- and starch-derivative-based microparticles in the period 2019–2023.

Polymer	Preparation Method	Active Substance	Reported Results	Reference
2-hydroxyethyl starch	Sonication method	Ketoprofen, Ofloxacin	Drug encapsulation efficiency 40–54%; sustained drug release at pH 1.2, 5.4, and 6.8; high efficiency against *E. coli* and *Bacillus cereus*.	[48]
Freeze-dried potato starch	Freeze-drying	Resveratrol	Starch microparticles, providing limited photo and thermal drug degradation during storage.	[51]
Maize starch	Molecular self-assembling	Dye model molecules	Monodispersed microparticles with size 0.2–5.0 µm; production yield over 70%; drug delivery to the intestines.	[102]
Crosslinked retrograded starch	Ionotropic gelation	Ketoprofen	Spherical microparticles with high yield (>78%) and encapsulation efficiency (72%). Sustained colonic drug delivery and good mucoadhesive properties.	[103]
Crosslinked starch	Emulsification, crosslinking	Clotrimazole	Sustained drug-release profile—34.31% clotrimazole released for 60 min, following zero-order kinetics and non-Fickian diffusion.	[104]
Cassava starch	Freeze-drying	*S. guianensis* essential oil	Increased stability of the essential oil; prolonged larvicidal activity; low toxicity of the microparticles on zebrafish embryos.	[105]
Debranched lentil starch	Complex coacervation	Rutin	Modified drug release from the microparticles; starch with higher molecular weight was beneficial for slower release of rutin.	[106]
Retrograded starch	Ionotropic gelation	5-fluorouracil	Targeted release of drug-loaded chitosan nanoparticles in the colon, tested in vivo on mice after oral administration.	[107]
Starch	Spray-drying	*Camelia sinensis* extract	Spray-dried microparticles with production yield 55–58%, encapsulation efficiency 60–93% and drug loading 65–84%; high antioxidant activity.	[108]
Corn and tapioca starch	Spray-drying	Bovine serum albumin	Spray-dried microparticles with protein loading 1–5% and entrapment efficiency 94–125%.	[109]
Starch, maltodextrin	Freeze-drying	Tannin extracts	Drug encapsulation efficiency 27–65%; drug loading 15–30%; in vitro burst release pattern in acetate buffer.	[110]
Carboxymethyl starch	Complex coacervation	Bovine serum albumin	Microparticles with oval-shape morphology and rough and porous surfaces; controlled release with limited burst effect, following zero-order kinetics.	[111]
Carboxymethyl starch	Ionotropic gelation, freeze-drying	Drug-free microparticles	Improved hydrophilic properties; substantially improved fluid absorption and swelling properties; improved clotting efficiency and hemostatic effect.	[112]
Starch/zein	Molecular self-assembling	Fucoxanthin	Starch-based carrier effectively protected fucoxanthin against photodegradation and oxidation; in vitro-controlled drug release.	[113]
Taro succinylated starch	Spray-drying	Pomegranate seed oil	Encapsulation efficiency of 1.09 ± 0.41%; highest amount of pomegranate seed oil was released under intestinal conditions.	[114]
Unhydrolyzed quinoa starch	Emulsification	Drug-free microparticles	Spherical, ellipsoidal in shape microparticles with the specific surface area of 1.676 m^2^/g; uniform size distribution with mean particle size of 28.5 μm.	[115]
Hi-maize resistant starch	Ionotropic gelation	Nisin	Drug protection and controlled release; effective inhibition of the growth of *C. tyrobutyricum*.	[116]
Maltodextrin	Spray-drying	Green tea extract catechins	Mean particle size of 26 μm; entrapment efficiency of 63%; improved thermal and pH stability of epigallocatechin gallate and gallocatechin gallate.	[117]
Maltodextrin, Inulin	Spray-drying	Flavonoid model drug	Microparticles showed excellent redispersibility and aerodynamic performance, suitable for inhalation administration.	[118]
Maltodextrin, whey protein	Spray-drying	α-Tocopherol, CoQ_10_	Distorted particle shape; high retention of the core material (89.6–97.4%) and antioxidant activity during storage for 35 days.	[119]
Maltodextrin, sweet potato starch	Spray-drying	*Lactiplantibacillus plantarum* (probiotics)	Materials used promoted greater resistance to *Lactiplantibacillus plantarum*; probiotic showed counts above 8.0 log CFU/g in the gastrointestinal tract.	[120]
Maltodextrin	Spray-drying	Polyphenols from *Lippia graveolens*	Microcapsules with spherical shape and particle size between 2 and 12 μm; high drug stability (85%); high antioxidant activity.	[121]
Maltodextrin, Arabic gum	Spray-drying	Lavender oil, Peppermint oil	Microcapsules with high yield 71–84%, mean diameter 2.41–5.99 µm, and total oil content of up to 10.80%.	[122]
γ-CD/hydroxypropyl-β-CD	Complexation	Nepafenac	Increased drug levels in the posterior eye segment after topical administration; non-irritating and non-toxic; high permeation through bovine sclera.	[123]
Carboxymethyl β-CD	Coacervation	Insulin	Increased drug stability in gastric environment; paracellular transport across Caco-2 cell; long-acting and stable hypoglycemic effect.	[124]
Methyl-β-CD, hydroxypropyl-β-CD	Freeze-drying Spray-drying	Quercetin	Nasal microparticles for nose-to-brain delivery; rapid in vitro dissolution and permeation; enhanced ex vivo transport across rabbit nasal mucosa.	[125]
Hydroxypropyl- β-CD	Spray-drying	Quercetin	Aerodynamic characteristic for delivery it in the alveolar region; anti-proliferative efficacy towards A549-adenocarcinomic-alveolar epithelial cells.	[126]
Thiolated hydroxypropyl-β-CD	Spray-drying	Budesonide	Nasal drug delivery; average diameter of 3.24 µm; prolonged mucosal residence time in vitro on freshly excised porcine nasal mucosa.	[127]
β-CD	Supercritical antisolvent co-precipitation	Albendazole	Particle mean diameter 0.45–1.4 μm; increased drug dissolution rate attributed to a synergic effect between the microparticle components.	[128]
β-CD	Emulsification	Meloxicam	Microparticles for periodontal pocket delivery; extended drug release up to 7 days with Fickian diffusion	[129]
β-CD	Emulsification	Celecoxib	Particle size 50–238 µm; entrapment efficiency 68–92%. Initial delayed release in stomach followed by fast release at colonic pH.	[130]

CD—cyclodextrin.

**Table 3:** Microspheres of cellulose and its derivatives.

Polymer	Solvent	Disperse Medium	Technique	Solidification	Size (µm)	Reference
Cellulose	NaOH/urea solution	Oil	Dispersing	HCl precipitation	10	[152]
Cellulose	NaOH/thiourea solution	Oil	Dispersing	HCl/CaCl_2_ precipitation	100	[153]
Cellulose acetate	CH_2_Cl_2_	Water	Dispersing	Solvent evaporation	100	[154]
Cellulose acetate	Ethyl acetate—methanol	Water	Dispersing	Solvent evaporation	1–10	[155]
Cell. acetate butyrate	CH_2_Cl_2_	Water	Dispersing	Acetic acid precipitation	100	[156]
Cellulose xanthate	NaOH solution	Benzene	Dispersing	Acetic acid precipitation	100	[157]
Cellulose xanthate	NaOH solution	Chlorobenzene	Dispersing	Temperature 90 °C	10–300	[158]
Cellulose	DMA/LiCl	-	Dripping	Alcohol precipitation	500	[159]
Cellulose acetate	Acetone/DMSO	-	Dripping	NaOH precipitation	900	[160]
Cellulose carbamate	NaOH solution	-	Jet cutting	H_2_SO_4_ precipitation	500	[161]
Cellulose	NaOH solution	-	Rotating	H_2_SO_4_ precipitation	500	[162]
Cellulose	NaOH/urea solution	-	Rotating	HCl precipitation	300	[149]

DMA: dimethylacetamide; DMSO: dimethylsulfoxide.

## Abbreviation

CD	Cyclodextrin
CDI	Carbonyldiimidazole
CMC	Carboxymethyl cellulose
DE	Dextrose equivalent
DP	Degree of polymerization
DPGDA	Dipropylene glycol diacrylate
DSM	Degradable starch microspheres
EC	Ethylcellulose
EE	Entrapment efficiency
FTIR	Fourier-transform infrared
HLB	Hydrophilic-lipophilic balance
HPC	Hydroxypropyl cellulose
HPMC	Hydroxypropyl methylcellulose
MC	Methylcellulose
MD	Maltodextrin
O/O	Oil-in-oil
O/W	Oil-in-water
STMP	Sodium trimetaphosphate
STPP	Sodium tripolyphosphate
W/O	Water-in-oil
W/W	Water-in-water
